# P-1768. Evaluation of Pharmacist-Driven Automatic 5-day Stop Dates for Ceftriaxone in Respiratory Tract Infections

**DOI:** 10.1093/ofid/ofae631.1931

**Published:** 2025-01-29

**Authors:** David T Adams, Satwinder S Kaur

**Affiliations:** Texas Health Harris Methodist Hospital Fort Worth, Fort Worth, Texas; Texas Health Harris Methodist Hospital Fort Worth, Fort Worth, Texas

## Abstract

**Background:**

Despite 2019 ATS/IDSA CAP guideline support for a 5-day treatment duration, compliance with these recommendations remains suboptimal. Patients that receive extended courses of antibiotics are at increased risk for adverse outcomes, longer hospitalization stays, and development of antimicrobial resistance. The objective of this study was to compare hospital length of stay (LOS) and length of therapy (LOT) before and after a pharmacist-driven automatic stop date protocol for ceftriaxone for CAP and COPD exacerbations.

**Methods:**

A retrospective cohort study conducted at Texas Health Harris Methodist Hospital Fort Worth evaluated patients before June 2023, and after November 2023. Patients were excluded if treatment indications were for non-respiratory indications, received ≤2 ceftriaxone doses, admitted into the ICU, LOS ≥10 days, or if placed on hospice within 72 hours of admission. Pharmacists then adjusted ceftriaxone orders to 5 total days for CAP and COPD exacerbations. The primary outcome was hospital LOS and LOT. Secondary outcomes included 30-day all-cause in-hospital mortality and 30-day hospital readmission due to infections.

**Results:**

295 patients were included in the final analysis with 145 patients in the pre- and 150 patients in the post-group. After implementation of the protocol, a statistically significant reduction in hospital LOS was observed in the post-group (4 days vs 5 days, *p* < 0.0001). There was also a statistically significant reduction in median LOT for CAP and COPD (5 days vs 7 days, *p* < 0.0001). No increase in 30-day all-cause mortality (0% vs 4%, *p* =0.012) or 30-day hospital re-admission rates due to infections (3% vs 8%, *p* =0.033) were observed in the post- compared to the pre-group.

**Conclusion:**

Based on the results of this study, implementation of a pharmacist-driven automatic 5-day stop date protocol for ceftriaxone is an effective antimicrobial stewardship initiative that can help increase compliance to guideline recommendations for CAP and COPD while also decreasing hospital LOS. Additionally, this initiative was not associated with increased rates of mortality or hospital re-admissions compared to longer durations and influenced discharge antibiotic prescription habits.

**Disclosures:**

**All Authors**: No reported disclosures

